# Climate Modelling Shows Increased Risk to *Eucalyptus sideroxylon* on the Eastern Coast of Australia Compared to *Eucalyptus albens*

**DOI:** 10.3390/plants6040058

**Published:** 2017-11-24

**Authors:** Farzin Shabani, Lalit Kumar, Mohsen Ahmadi

**Affiliations:** 1 Ecosystem Management, School of Environmental and Rural Science, University of New England, Armidale, NSW 2351, Australia; lkumar@une.edu.au; 2 Department of Natural Resources, Isfahan University of Technology, Isfahan 84156-83111, Iran; ahmadim.bio@gmail.com

**Keywords:** *Eucalyptus albens*, *Eucalyptus sideroxylon*, species distribution models, range, climate change, GIS

## Abstract

**Aim:** To identify the extent and direction of range shift of *Eucalyptus sideroxylon* and *E. albens* in Australia by 2050 through an ensemble forecast of four species distribution models (SDMs). Each was generated using four global climate models (GCMs), under two representative concentration pathways (RCPs). **Location:** Australia. **Methods**: We used four SDMs of (i) generalized linear model, (ii) MaxEnt, (iii) random forest, and (iv) boosted regression tree to construct SDMs for species *E. sideroxylon* and *E. albens* under four GCMs including (a) MRI-CGCM3, (b) MIROC5, (c) HadGEM2-AO and (d) CCSM4, under two RCPs of 4.5 and 6.0. Here, the true skill statistic (TSS) index was used to assess the accuracy of each SDM. **Results:** Results showed that *E. albens* and *E. sideroxylon* will lose large areas of their current suitable range by 2050 and *E. sideroxylon* is projected to gain in eastern and southeastern Australia. Some areas were also projected to remain suitable for each species between now and 2050. Our modelling showed that *E. sideroxylon* will lose suitable habitat on the western side and will not gain any on the eastern side because this region is one the most heavily populated areas in the country, and the populated areas are moving westward. The predicted decrease in *E. sideroxylon’s* distribution suggests that land managers should monitor its population closely, and evaluate whether it meets criteria for a protected legal status. **Main conclusions:** Both *Eucalyptus sideroxylon* and *E. albens* will be negatively affected by climate change and it is projected that *E. sideroxylon* will be at greater risk of losing habitat than *E. albens*.

## 1. Introduction

In Australia, *Eucalyptus albens* (white box) used to be a dominant species found in large continuous forests stretching from southern Queensland through Western New South Wales (NSW) and Victoria’s Woodlands [1], and have been neglected in terms of conservation and are now listed as an ecologically endangered species. The habitats of the species are endangered by grazing, soil degeneration, invasion of weeds, and increased salinity [2]. *Eucalyptus sideroxylon* (red ironbark) is another dominant Australian species, and its distribution is wide but sporadic: southeastern Queensland, widespread on the western slopes and plains of NSW south into north-central Victoria. Based on a recent published document by the Office of Environment and Heritage [3] *Eucalyptus sideroxylon* and *E. albens* have common regional habitats [2]. What does the future hold for *Eucalyptus sideroxylon* and *E. albens*? Research in a number of disciplines indicates that over the next century, climate change may become the major factor causing the extinction of species [4]; directly as a single factor, as well as indirectly in conjunction with other factors [5].

The use of the current distribution of a species has become common in species climate modelling [6]. Despite the limitations inherent in correlative modelling [7], the projections of ‘envelope’ models have been used as the basis for estimating the likelihood of the extinction or shift of species under climate change in numerous taxa and in many global regions [4,8,9,10,11,12,13]. For example, studies have considered the potential of climate change impact on the production of maize [14], cotton (*Gossypium*) [15,16,17], *Fusarium oxysporum* f. spp. [18], *Triticum aestivum* [19], *Phoenix dactylifera* L. [8,20,21], *Rhynchospora alba* (white beak-sedge), *Salix herbacea* (dwarf willow) [22], *Triadica sebifera* [23], *Elaeis guineensis* Jacq. [24], *Acer campbellii* [25], *Puccinia sorghi* [26], *Neoleucinodes elegantalis* [13] and *Aspergillus niger* [27].

The last decade has seen the rise of species distribution models (SDMs) as a method of projecting distributions under future climate change [28]. Researchers have increasingly questioned certain approaches and assumptions employed in SDM studies [29,30]. Some of the more prominent queries include:

- Whether the use of solely climate variables is sufficient for projecting future distributions across large areas [31];
- Arbitrariness in the selection of particular nonclimatic variables, and the number of these chosen, and when these are included in the SDM approach, making comparative analyses with similar studies under other approaches difficult [32]. However, a proposal has been made that the adoption of a standard basic configuration of valid ecological variables would enable the testing of SDM ecological assumptions, comparisons with other sets of predictors for similar biophysical processes, as well as providing a basis for the choice of the scale of resolution [33];
- Rating model performance with an independent set of validation data;
- Whether validation by means of an independent set of distribution data is a viable alternative for accuracy of independent data;
- Which techniques consistently outperform others; and
- Whether native, exotic or a combination of both records produces greater modelling efficiency.

**Figure 1:**
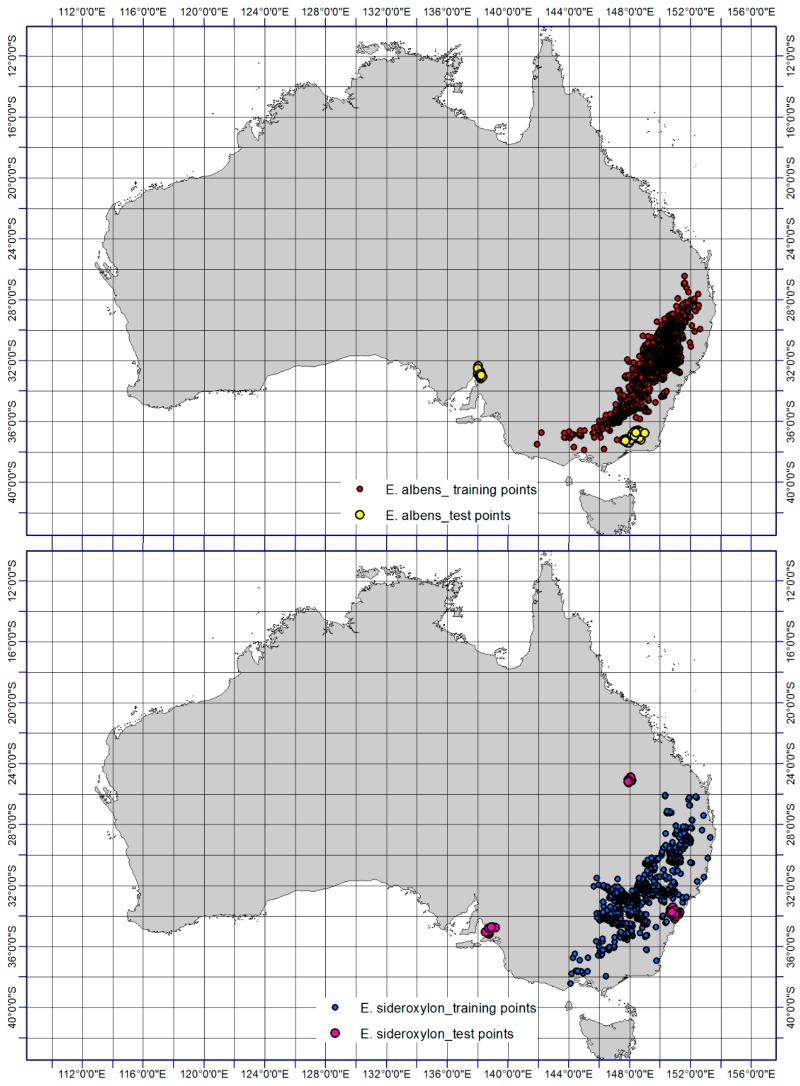
Distribution records (test and training points) of *Eucalyptus sideroxylon* and *E. albens*. Training points were used to develop climate suitability models while test points were kept aside for model validation purposes.

The Office of Environment and Heritage [3], an Australian government agency that protects and conserves the natural environment, has recently documented that *Eucalyptus sideroxylon* and *E. albens* have common regional habitat and both need fertile soils and slopes. Based on the current and historical occurrence records of both species, it is likely that there are some ecological similarities between these two species as shown in Figure 1. We believe that the possible future range separation of the two species that currently have partially or largely overlapping range could provide a clear view of the extent and nature of likely change in distribution for these two species. Our study set out to model the extent and direction of range shift of *Eucalyptus sideroxylon* and *E. albens* in Australia by 2050 through ensemble model of four machine-learning SDMs including (i) generalized linear model (GLM), (ii) MaxEnt, (iii) random forest (RF), and (iv) boosted regression tree (BRT), four GCMs including (a) MRI-CGCM3, (b) MIROC5, (c) HadGEM2-AO and (d) CCSM4 under two RCPs scenarios of 4.5 and 6.0. An ensemble method was used to combine the four SDM outputs into a single layer for each of the species and databases. Our primary goals were to (1) predict how the distributions of our chosen species might change over the next 80 years, given the expected changes in climate, and thereby assist land managers with ensuring sufficient habitat provision to maintain populations of these species; and (2) evaluate how the choice of particular SDMs, GCMs, and climate scenarios impact these projections, and thereby provide guidance to forest managers on the strengths and weaknesses of different approaches.

## 2. Methodology

### 2.1. Distribution Records and Species Phenomena

A variety of sources were used for the compilation of distribution data. Global data was obtained from the Global Biodiversity Information Facility [34], Atlas of Living Australia [35], and related literary sources. As this information might have suffered spatial biases in sampling effort, resulting in overfitting of spatial models in areas with clumping of presence points [36] we performed a spatial filtering procedure to account for spatially biased records [37]. The georeferenced occurrence data of each individual grid cell was converted to a single unit (10 km by 10 km) equaling 1 in ENMTools [38]. Therfore, presence points were filtered to only single points within a 10-km distance from others and the number of records for a single grid-cell does not impact on model projections and the statistical testing of performance. The dataset comprised all native and exotic distribution records, as the study was not concerned with the effects of the inclusion of one or both of these categories of records on the performance of a technique in projecting suitability of climate. The distribution records of *Eucalyptus albens* (*N* = 6250 occurrences) and *E. sideroxylon* (*N* = 2541 occurrences) are illustrated in Figure 1. 

The Australian *E. albens* (white box) woodlands have been neglected in terms of conservation and are now listed as an endangered species [39]. The habitats of the species are endangered by grazing, soil degeneration, invasion of weeds, and increased salinity [40]. *E. albens* is a dominant or co-dominant tree that can usually be found in mixed wood evergreen forests on fertile volcanic soils throughout southeastern Australia; the species reproduces primarily by seed, and recovers quickly after natural disturbance (e.g., fire), but is sensitive to prolonged drought. *Eucalyptus sideroxylon* (red ironbark) is a tall evergreen tree with a single trunk, and black bark with deep fissures on both trunks and branches. 

### 2.2. Species Distribution Modelling

The two species were modelled using the four bioclimatic modelling techniques: GLM [41], MaxEnt [42], BRT [43] and RF [44]. We used Biomod framework, by which we could implement all models in a controlled environment and combine them to a final model called an ‘ensemble’ model. The modelling approach was done using the ‘biomod2’ package [45] in R v. 3.2.5 [46].

#### 2.2.1. GLM

In GLM, the maximum likelihood of the parameters was estimated using iterative linear regression weighting, with an exponential distribution of observations and a linear transformation of the systematic effects. The variable of response was linked to the combination of linear and quadratic explanatory variables using parametric functions. The models were fitted with a standard polynomial approach and an automatic stepwise model selection according to the Akaike Information Criterion (AIC).

#### 2.2.2. MaxEnt

Using MaxEnt, we defined a background geographical dataset [47], to compare the climate of a sampled reference set of grid cells with the climate of grid cells indicating a presence of the species. The composition of the background dataset significantly influences the results of the model [48] and it should represent the full environmental range of the species in the study area [49]. For the MaxEnt algorithm, we compared the complexity of interactions between presence locations and related variables to those with similar interactions to the background geographical locations, in order to find the maximum entropy probability distribution nearest to uniformity, subject to the limitations of the observed spatial distributions and related environmental variables. Through minimizing the relative entropy between presence data and background point data using this method, the probability distribution is optimized [42].

#### 2.2.3. RF

RF has been rated as one of the most accurate classificatory or regression tree-based models [44]. In RF, the bootstrap aggregation performs a selection of many subsamples from the data and, using a bagging algorithm, generates many decorrelated regression trees [44]. Thus, each tree is made dependent on independently sampled random vector values, which exhibit similarity of distribution for all trees in the forest [44]. We chose RF because of its grouping of unpruned regression trees, formed from training data bootstrap samples, and its selection of random features in the tree induction process. A majority, or averaging, of predictions in the ensemble generates the prediction [50]. Predictions of model errors and rating of importance of variables is based on out-of-bag observations in each tree. Predictions of the grown decision trees are averaged, as in an ensemble approach. To fit RF models we used the ‘RandomForest’ package [51]. 

#### 2.2.4. BRT

In the BRT model, we used the same MaxEnt background area to the modelling and all eight predictors, fitting many combinations (decision trees) iteratively, to achieve a performance enhancement. Using two multiple regression tree algorithms based on a binary division into rectangles of predictor space it correlates predictor responses to expanses with the most homogeneous responses, as well as the additional boosting procedure of merging fitted trees for enhanced accuracy. The ‘GBM’ package [52] in R environment v 3.1.2 [46], with a supplementary setting code recommended by Elith, Leathwick [43], was used to fit our BRT models. 

### 2.3. Bioclim Variables, Background Data, Test Points & True Skill Statistic (TSS)

We focused on climatic variables obtained from WorldcClim database [53]. This dataset comprises 19 climatic variables that are temperature or rainfall related variables. Because of the inherent high correlation between climatic variables, we limited them to those describing absolute values of temperature and humidity (e.g., annual mean temperature [°C], bio1, and annual precipitation [mm], bio12) and variation in these climatic parameters through the year (e.g., temperature seasonality, bio4, and precipitation seasonality, bio15). We also used the jackknife analysis method and correlation coefficient results for each species using the Pearson technique to make sure that the major chosen variables (bio1, bio4, bio12 & bio15) had high contribution with low correlation (*R*^2^ < 0.5) were chosen for all approaches. 

In creating background data, to compensate for the likelihood of fewer records from more recently invaded areas, as well as those with poorer sampling, prominence was given to those areas further away from heavily invaded areas. However, without records on survey efficiency and duration, a differentiation between unsuitable and undersampled areas is impossible, and the compensation cannot distinguish between the two categories. To calculate weighting surface, the Gaussian kernel method was applied in ArcGIS using the default values’ standard deviations to calculate the number of weighted records in a selected geographical environment for each cell for Australia and was divided by the weighted number of terrestrial cells of the same region. This eliminates coastal edge effects. Thereafter, the resulting grid was scaled to produce a maximum value of 20 and a minimum of 1, thus excluding any extreme values. This method was advised by Elith, Kearney [49] for the purpose of minimizing bias against sparsely sampled records from those of densely sampled areas. The kernel density layer of each species and Hawths Analysis Tools generated the background points for the entire research area that was used for training. Thus, all SDMs shared the same background data for both species to facilitate evaluating comparative performance. 

For this research, we purposefully selected testing points in those regions that had geographic outliers. We believe that this gives a better basis for validation of the model compared to selecting test points from regions of high occurrence data. 

We used the area under the receiver operating characteristic curve (AUC; ranging from 0.5 = random to 1 = perfect discrimination) and true skill statistic (TSS) to compare prediction performance of different types of SDMs. TSS expresses sensitivity + specificity − 1 [54]; sensitivity indicates the proportion of observed presences predicted, thus quantifying errors of omission; specificity indicates the proportion of observed absences predicted, thus quantifying errors of commission. TSS is an appropriate tool for the measurement of accuracy among model results [55].

The importance of the variables, and thus their contribution to each model in biomod modelling approach, was examined regarding Pearson rank correlation between standard predictions and those based on a permutation randomized five times for each variable separately [45]. For each variable, we then averaged variable importance across all implemented models. Additional information on the modelling approaches and the Bio predictors are found in the supplementary material.

## 3. Results 

### 3.1. Model Validation & Models of Current Distribution

**Figure 2:**
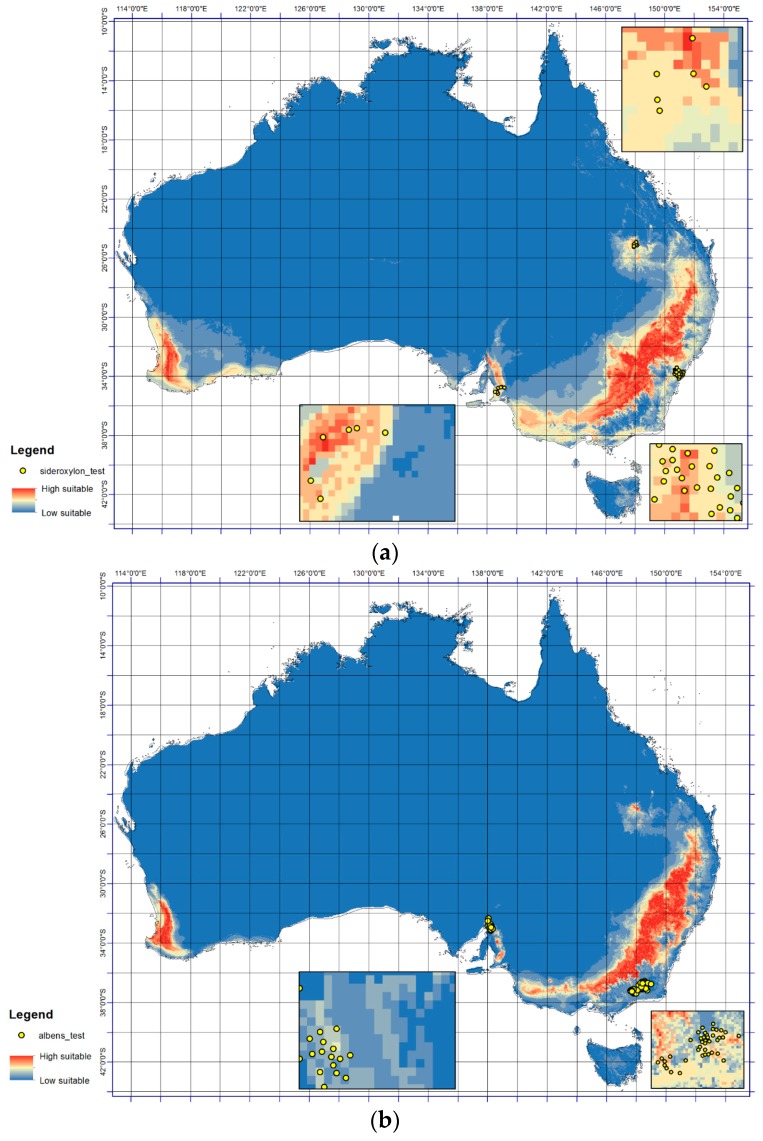
The testing point response surfaces at continental and enlarged scale for (**a**) *E. sideroxylon* and (**b**) *E. albens*. The lowest (most unsuitable) predicted occurrence probability is indicated by the cool colors and the optimal range by the warmer color.

Notable consistency was detected in the results (Figure 2a,b) for the presence of *E. sideroxylon* and *E. albens* at a continental scale when compared with presence records (Figure 1). For example for *E. albens*, the models predict large regions from 36° S to 38° S and 140° E to 148° E (Figure 2b) as well as from 32° S to 36° S and 148° E to 149° E will become optimal (Figure 2b), which displays consistency with the occurrence data. However, large regions in western Australia between 32° S from 35° S and 115° E to 117° E (Figure 2a,b) are predicted as optimal for both *E. sideroxylon* and *E. albens* occurrence, despite no current presence (Figure 1). The TSS values for *E. sideroxylon* and *E. albens* in ensemble were 0.66 and 0.78 respectively (Table 1). In this regard, it must be noted that probability class patterns in environmental space do not necessarily reflect their spatial extent in predictions of geographical species distributions. 

### 3.2. Variables Contributions on Response Surfaces

**Figure 3:**
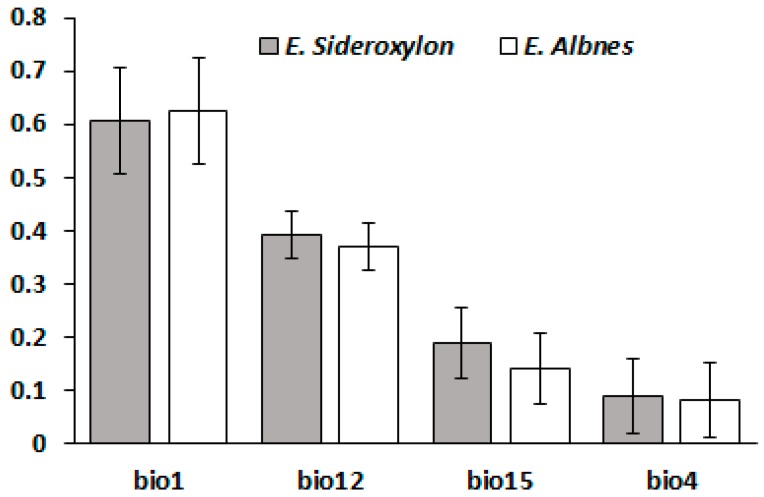
Variables contributions on response *Eucalyptus sideroxylon* and *albens*. bio1 (Annual Mean Temperature [°C]), bio4 (Temperature Seasonality [°C]), bio12 (Annual Precipitation [mm]), and bio15 (Precipitation of Wettest Quarter).

Figure 3 shows climatic variables contributions on response surfaces for *E. sideroxylon* and *E. albens* and it is apparent that Bio1 (Annual Mean Temperature [°C]) and Bio12 (Annual Precipitation [mm]) had the greatest impact on the response surfaces of both species. It should be noted that the impacts of all climatic variables on the response surfaces for both species were similar (Bio1 > Bio12 > Bio15 (Precipitation of Wettest Quarter) > Bio4 (Temperature Seasonality [°C])) (Figure 3). 

### 3.3. Range Gain, Loss or As Is 

**Figure 4:**
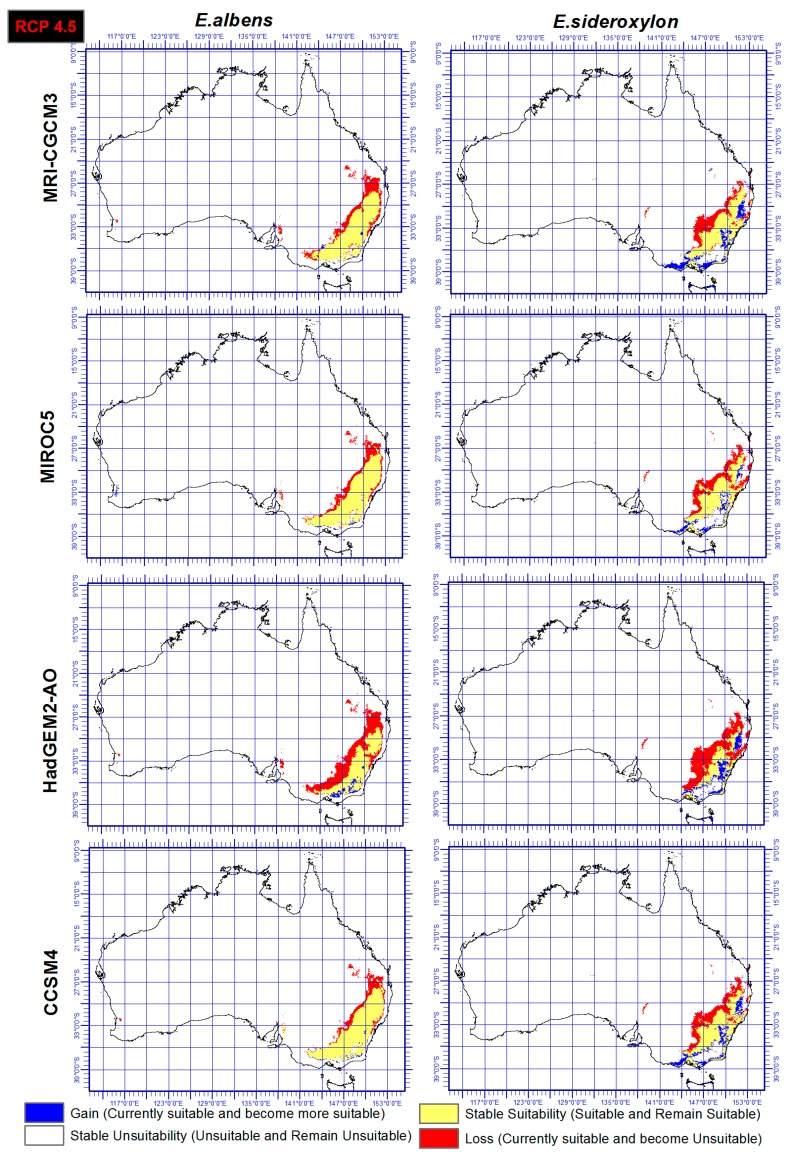
Ensemble output of four SDMs, under RCP 4.5 and four GCMs indicating range gain, loss or as is of *Eucalyptus sideroxylon* and *E. albens* by 2050.

**Figure 5:**
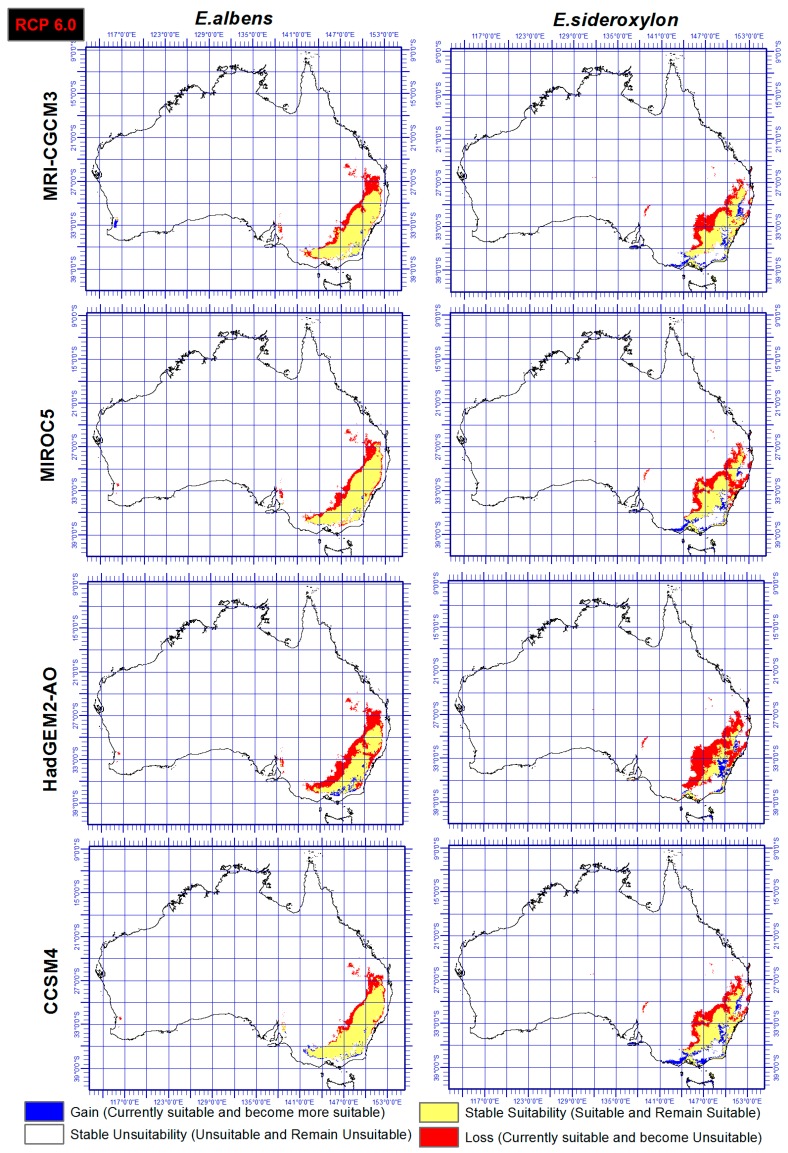
Ensemble output of four SDMs, under RCP 6.0 and four GCMs indicating range gain, loss or as is of *Eucalyptus sideroxylon* and *E. albens* by 2050.

Range shift of both *E. sideroxylon* and *E. albens* were identified through the ensemble output of four SDMs, under both RCP and four GCMs. Results showed that *E. albens* and *E. sideroxylon* will lose large areas of their current range by 2050 (Figure 4 and Figure 5). Our results showed that *E. sideroxylon* is projected to gain range in eastern and southeastern Australia. Some areas were also projected to remain suitable for each species between now and 2050. In line with this matter, the ensemble output of four SDMs under GCM of HadGEM2-AO showed a greater range loss than the other three GCMs (MRI-CGCM3, MIROC5, and CCSM4) for both *Eucalyptus* species. Our projections also showed that there is a possibility that *E. albens* gains range in areas that are completely unsuitable or *E. sideroxylon* in the southeastern regions between 142 to 147° E, and 34 to 38° S. 

## 4. Discussion

The aim of this study was to identify the extent and direction of range shift of *Eucalyptus sideroxylon* and *E. albens* in Australia by 2050 through ensemble model of four SDMs, four GCMs, under two RCPs. In our research, a number of databases were used to obtain data, while presence data was collected in the field and may thus represent a local microclimate of suitability for the species, which is not reflected in the coarse climate data. This can result in unrealistically optimistic predictions of climate suitability, in that specific areas of calibration may exhibit more climatic variation within a 10′ pixel, than variations found in Australia [33]. 

Analyzing the results of the four SDMs individually showed some differences between method projections, which is due to each one functioning slightly differently and, in line with this matter, Shabni et al. [55] have documented that it may be safer to use an ensemble of models. We also note that the comparison of the individual SDM to an ensemble approach showed that there was a better agreement between the ensemble outputs under different GCMs. This finding is in line with Araújo and New [56], who have recommended that using ensemble forecasting has clear advantages over single model forecasts.

In a comparison of the four GCM outputs for *Eucalyptus albens*, we see that under the HadGEM2-AO GCM, there is a greater loss in area compared to the other three GCMs. Similar trends also were seen for *Eucalyptus sideroxylon* under HadGEM2-AO GCM, while the CCSM4 GCM projected the least loss for both species and these could be based on the different functions they use. However, it should be mentioned that there is substantial confidence that climate models provide credible quantitative projections of future climate alteration, mainly at continental scales and above as they are heavily based on accepted physical principles [57].

In this study, two different RCPs were utilized and the output showed that there is a general agreement between both RCPs (4.5 and 6.0) and RCP 6.0 projected just slightly higher losses for both species especially under HadGEM2-AO GCM. However, it is not yet possible to determine which estimates of the climate change and RCPs feedbacks are the most reliable [57].

**Figure 6:**
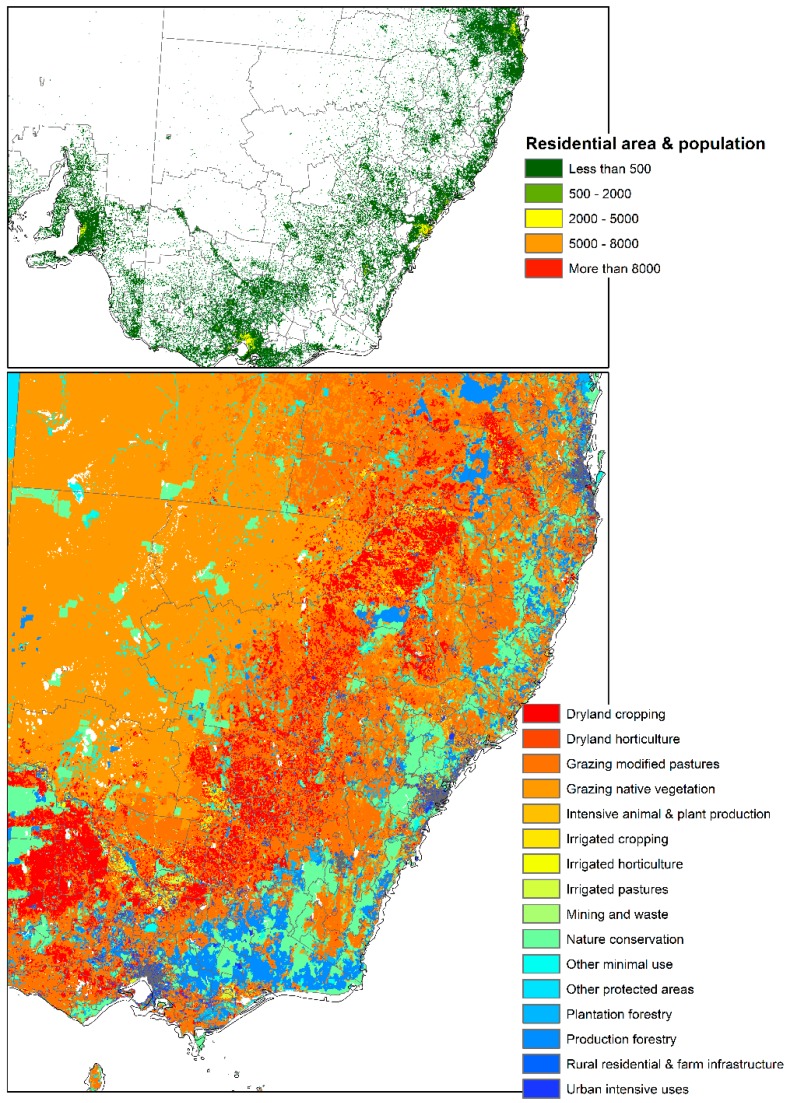
Current location of residential areas and its population, land use type and land management of eastern side of Australia. Data obtained from *Australian Bureau of Agricultural and Resource Economics and Sciences* available at: http://www.agriculture.gov.au/abares.

This study identified the extent and direction of range shift of *Eucalyptus sideroxylon* and *E. albens* in Australia by 2050 through ensemble model of four SDMs, four GCMs, under two RCPs. The results presented in Figure 4 and Figure 5 indicate disappearing and separating ecological environments of the two species that currently share their range. The total area that has common suitability between *Eucalyptus sideroxylon* and *E. albens* is 139,410,000 hectares currently and 131,010,000 hectares in 2050 under RCP 4.5 and 127,230,000 hectares under RCP 6.0. From this, we see that the areas suitable for both species to occur together decreases into the future. Conversely, the suitable area that is not common between the two species at current time is 21,130,000 hectares, and in 2050 it is 71,750,000 hectares under RCP 4.5 and 45,540,000 hectares under RCP 6.0 (Figure 4 and Figure 5). This shows that the species are separating in their habitat. In line with this importance, our results also indicate that *E. sideroxylon* will gain range in southeastern Australia; however, this might not happen as predicted, since the majority of the current Australian population, residential areas, infrastructure and industrial/agricultural regions are within these regions as shown in Figure 6. 

For the above reasons, the predicted decrease in *E. sideroxylon’s* distribution suggests that land managers should monitor its population closely, and evaluate whether it meets the criteria for a protected legal status. Our projections also show that *E. albens* will have stable range between now and the future. Here, the provided maps deliver an opportunity to engage with communities and stakeholders about values into the future and the possible requirement to consider adjusting objectives.

Variations in results and apparent differences in behaviour were apparent in our testing of different methods and datasets, indicating that the question of appropriateness needs to be answered ecologically and/or physiologically, as much as statistically. Besides, it is widely documented that different GCMs tend to model different projections and even produce contradicting outputs, primarily due to different assumptions, simplifications or parameterizations employed by different GCMs [58,59]. However, an ensemble of multiple GCMs provides an opportunity to define the model spread of possible future climate projections. In line with this matter, it has been documented that bias correction algorithms are suitable tools to force all GCMs to have similar statistics [60]. Furthermore, statistical bias correction algorithms can correct biases in different GCMs due to uncertainties in the parameterizations of unresolved processes [61]. 

In terms of execution, the choices exceeded those we have demonstrated. As an example, MaxEnt has an inbuilt ability to predict absence beyond the data range. To effectively analyze efficiency in extrapolation and forecasting, a wider range of tests is essential, including evaluating predictive ability into novel environmental combinations. An understanding of the workings and performance of specific models, and the formulation of criteria of evaluation that are suitably matched to the associated questions, facilitates effective decision making in terms of the modelling approach [58,59]. For more clarification, refer to Elith and Graham [58]. Thus, a comparative knowledge of performance efficiency and limitations of available SDM methods should precede exercises in the modelling of species distribution for these to be considered scientifically valid.

Comparing predictions of both species under both RCPs (Figure 4 and Figure 5) shows short distance, major habitat changes affecting the potential suitability of local habitats. Where climate change impacts negatively on species distributions, survival is dependent on local habitats, and similarly, where climate conditions offer support for a species, expansion must be conditional on the suitability of other variables. Future research must investigate the integration of other factors that can potentially increase or reduce limitations on species distributions. Factors such as land cover, biotic interactions, direct effects of CO_2_, alterations to related biotic interactions, and mechanisms of dispersal and propagation of dispersal must be included in species distribution modelling. 

## 5. Conclusions

Here, we identified the extent and direction of the range shift of *Eucalyptus sideroxylon* and *E. albens* in Australia by 2050 through an ensemble model of four SDMs, four GCMs, under two RCPs. The predictors chosen for each species were: annual mean temperature (°C), temperature seasonality (°C), annual precipitation (mm), and precipitation seasonality. Our results showed that there is an eastward shift in the species, with the western part losing habitat suitability. Overall, these species are losing suitability and gaining slight suitability on the eastern side. However the eastern side is the most heavily populated region in the country, and the populated areas are moving westward. This is a competition that the humans will win, thus further decreasing suitable habitats for both species. Furthermore, we conclude that our results provided a clear view of the extent and nature of likely change in biodiversity of two important Australian *Eucalyptus*. We found that *E. sideroxylon* will be at greater risk of losing habitats than *E. albens* and thus land managers should monitor its population closely, and evaluate whether it meets criteria for a protected legal status. This species will be negatively affected by climate change and will find its suitable range in regions with high populations, residential areas, infrastructure and industry. 

There were some differences in the projection of suitable range for each species obtained by different GCMs. Differences in the outcomes from the different GCMs emphasize the uncertainties associated with the state of climate modelling. Greenhouse emission patterns and errors are inherent in bioclimatic modelling, and the fundamental value of models is arguably heuristic, rather than predictive. 

## Figures and Tables

**Table 1:** TSS value (testing, training) for *E. sideroxylon* and *E. albens* for four different SDMs.

	TSS (Testing, Training)			
	GLM	MaxEnt	RF	BRT
*E. sideroxylon*	0.66, 0.86	0.67, 0.88	0.64, 0.88	0.67, 0.88
*E. albens*	0.78, 0.92	0.79, 0.92	0.76, 0.92	0.78, 0.92

## Supplementary Materials

The following are available online at www.mdpi.com/2223-7747/6/4/58/s1.
