# Supplementary material for: Climate Modelling Shows Increased Risk to Eucalyptus sideroxylon on the Eastern Coast of Australia Compared to Eucalyptus albens
Source: Plants (Basel). 2017 Nov 24;6(4):58. doi: 10.3390/plants6040058 (PMC5750634; doi:10.3390/plants6040058)
Supplement: Supplementary file 1 [file plants-06-00058-s001.pdf]

Methodological information

Bioclimatic variables (Environmental variables)

Bioclimatic variables are derived from the monthly temperature and rainfall values in order to generate more biologically meaningful variables. These are often used in species distribution modeling and related ecological modeling techniques. The bioclimatic variables represent annual trends (e.g., mean annual temperature, annual precipitation) seasonality (e.g., annual range in temperature and precipitation) and extreme or limiting environmental factors (e.g., temperature of the coldest and warmest month, and precipitation of the wet and dry quarters). A quarter is a period of three months (1/4 of the year).

They are coded as follows:

BIO1 = Annual Mean Temperature
BIO2 = Mean Diurnal Range (Mean of monthly (max temp - min temp))
BIO3 = Isothermality (BIO2/BIO7) (* 100)
BIO4 = Temperature Seasonality (standard deviation *100)
BIO5 = Max Temperature of Warmest Month
BIO6 = Min Temperature of Coldest Month
BIO7 = Temperature Annual Range (BIO5-BIO6)
BIO8 = Mean Temperature of Wettest Quarter
BIO9 = Mean Temperature of Driest Quarter
BIO10 = Mean Temperature of Warmest Quarter
BIO11 = Mean Temperature of Coldest Quarter
BIO12 = Annual Precipitation
BIO13 = Precipitation of Wettest Month
BIO14 = Precipitation of Driest Month
BIO15 = Precipitation Seasonality (Coefficient of Variation)
BIO16 = Precipitation of Wettest Quarter
BIO17 = Precipitation of Driest Quarter
BIO18 = Precipitation of Warmest Quarter
BIO19 = Precipitation of Coldest Quarter

In this study, the predictors chosen for each species were: bio1 (annual mean temperature (°C)), bio4 (season of temperature (°C)), bio12 (annual mean precipitation (mm)), and bio15 (season of precipitation).

Jackknife, Background data, Hawths Analysis Tool

Through the **jackknife** analysis method avaiable in MaxEnt and the correlation of coefficient results by Pearson correlation technique for each species, the most important variables with low correlation ($R^2 < 0.5$) were selected and used in modeling approaches.
In order to create **background data** in terms of the possibility that there would be fewer records returned from areas of more recent invasions and areas that were poorly sampled, we gave prominence to those having less geographical proximity to others. However we note that

without records on survey effort in terms of time, one cannot differentiate between environmentally unsuitable and under-sampled areas, and that these adjustments will unavoidably confuse the two categories. To calculate weighting surface, the number of weighted records (Gaussian kernel method with a standard deviation of the default values in ArcGIS) in a selected geographical environment for each cell was divided by the weighted number of terrestrial cells in the specific geographical environment (to avoid edge effects along coasts). The resulting grid was then scaled to give a maximum of 20 and a minimum of 1, to exclude extreme values. This method of weighting is advocated by Elith, Kearney [1] to minimize bias favouring records from densely sampled areas over those from sparsely sampled areas.

Hawths Analysis Tool provides a suite of solutions for tasks common in spatial analysis. Specifically developed for ecological studies, these tools can be used in any application for analyzing spatial data. They extend (and in some cases simplify) core ArcGIS functionality that is not available out-of-the-box. The extension consists of more than fifty tools that cover a broad range of analysis types including analyzing, sampling, and editing vector as well as raster data, and tools for common operations in tables and CSV files. Some abilities of this tool is:

- Create Random Selection tool for selecting specified number or percentage of random features (points, lines, or polygons)
- Generate Random Points tool for generating a defined number of points over a specified area
- Create Vector Grid for generating a grid over a specified area and of a defined grid cell size
- Thematic Raster Summary for summarizing the frequency of cells of categories in a thematic raster layer by polygon
- Sum Values for calculating the total value of all/selected rows in a numeric field
- List Unique Values for listing all unique values in a selected field
- Delete Multiple fields for removing multiple fields from a table all at once
- Intersect Lines for generating point features at line intersections

The kernel density layer of species and the Hawths Analysis Tools were used for generating background points to be used for training purposes.

Modifications to settings: MaxEnt produces an index of suitability between 0 (unsuitable) to 1 (most suitable). For finding an optimal model for both species, we adjusted different settings in MaxEnt since default settings are not always the best [2]. For that reason, we made adjustments of different combinations of feature types and regularization multiplier (RM). First, we combined different sets of MaxEnt features (i.e. linear [L], quadratic [Q], product [P], threshold [T], and hinge [H]) (Table 3 and 4) with RM. The RM is used to control the number of parameters and consequently the complexity of the model [3, 4]. Models are very restricted when is used RM below 1 which is not well appropriate for world predictions, when RM is higher than 1, it may generate models with a broader potential distribution [5]. The 'fade-by-clamping' option in MaxEnt was selected to prevent extrapolations out of the environmental range [6]. The jackknife' technique and the percent contribution were used to estimate the predictive contribution / importance of different environmental variable. Response curves are generated by MaxEnt and we used only the ones that shows the relationships between predicted probabilities of presence for each species separately, considering the variation in each

environmental predictors. All responses curves were evaluated in terms of making biological sense, models without biological sense were not considered for further evaluations. For ranking the models' performance we evaluated the test sensitivity at 0% and 10% training Omission Rates (OR) [7, 8] and the AUC_{cv} (area under the receiver operating characteristic [ROC] curve) [9]. For calculate AUC_{cv} and OR, 10-fold cross-validation in MaxEnt were ran. To discriminate presence from background we used the AUC_{cv}. When AUC_{cv} value is 0.5 means that model predictions are not better than random; values below 0.5 are worse than random; between 0.5-0.7 indicate poor performance; between 0.7-0.9 indicate reasonable or moderate performance; and values higher than 0.9 shows high performance [10]. For the OR, the expected value of test omission rate at 0% training OR is 0, whereas at 10% training OR threshold it is 0.10; models show poor performance when those number are higher than expected omission rates [11]. The models were ranked based on 10% training OR, 0% training OR, and AUC_{cv}, respectively [4, 7, 12].

Reference list

1. Elith, J., M. Kearney, and S. Phillips, *The art of modelling range-shifting species.* Methods in ecology and evolution, 2010. **1**(4): p. 330-342.
2. Kumar, S., L. Neven, and W.L. Yee, *Evaluating correlative and mechanistic niche models for assessing the risk of pest establishment.* Ecosphere, 2014. **5**(7): p. 1-23.
3. Elith, J., et al., *A statistical explanation of MaxEnt for ecologists.* Diversity and distributions, 2011. **17**(1): p. 43-57.
4. Merow, C., M.J. Smith, and J.A. Silander, *A practical guide to MaxEnt for modeling species' distributions: what it does, and why inputs and settings matter.* Ecography, 2013. **36**(10): p. 1058-1069.
5. Phillips, S.J., R.P. Anderson, and R.E. Schapire, *Maximum entropy modeling of species geographic distributions.* Ecological modelling, 2006. **190**(3): p. 231-259.
6. Owens, H.L., et al., *Constraints on interpretation of ecological niche models by limited environmental ranges on calibration areas.* Ecological modelling, 2013. **263**: p. 10-18.
7. Liu, C., M. White, and G. Newell, *Selecting thresholds for the prediction of species occurrence with presence-only data.* Journal of Biogeography, 2013. **40**(4): p. 778-789.
8. Kumar, S., et al., *Assessing the global risk of establishment of Cydia pomonella (Lepidoptera: Tortricidae) using CLIMEX and MaxEnt Niche models.* Journal of Economic Entomology, 2015. **108**(4): p. 1708-1719.
9. Peterson, A.T., M. Papeş, and J. Soberón, *Rethinking receiver operating characteristic analysis applications in ecological niche modeling.* Ecological modelling, 2008. **213**(1): p. 63-72.
10. Peterson, A., et al., *Ecological Niches and Geogrpahical Distributions Princeton*. 2011, Oxford: Princeton University Press.
11. Boria, R.A., et al., *Spatial filtering to reduce sampling bias can improve the performance of ecological niche models.* Ecological modelling, 2014. **275**: p. 73-77.
12. Kumar, S., L.G. Neven, and W.L. Yee, *Assessing the potential for establishment of western cherry fruit fly using ecological niche modeling.* Journal of Economic Entomology, 2014. **107**(3): p. 1032-1044.

Methodological information
